# DKS26 Alleviates Ischemia-Reperfusion Injury-Induced Acute Kidney Injury by Stabilizing Vitamin D Receptors to Inhibit the Inflammatory Pathway of NF-κB P65

**DOI:** 10.3390/ijms26072985

**Published:** 2025-03-25

**Authors:** Luqun Liang, Yuanyuan Ruan, Xiong Yu, Wanlin Tan, Xiaoxiao Xu, Jing Jia, Jin Peng, Fangfang Wang, Yulin Peng, Yuting Chen, Lingling Liu, Bing Guo, Jiquan Zhang, Yuanyuan Wang

**Affiliations:** 1 Department of Pathophysiology, Guizhou Medical University, No. 6 Ankang Road, Guiyang 561113, China; 18385157974@163.com (L.L.); 18300854323@163.com (Y.R.); y2511093632@163.com (X.Y.); tanwanlin@gmc.edu.cn (W.T.); xx232r66@163.com (X.X.); 13985263693@163.com (J.J.); lydxdmm@126.com (J.P.); WFfang1234567@163.com (F.W.); 13142910112@163.com (Y.P.); 15902550037@163.com (Y.C.); 13158016661@163.com (L.L.); Guobingbs@126.com (B.G.); 2 Guizhou Provincial Key Laboratory of Pathogenesis and Drug Research on Common Chronic Diseases, Guizhou Medical University, No. 6 Ankang Road, Guiyang 561113, China; 3 State Key Laboratory of Functions and Applications of Medicinal Plants, College of Pharmacy, Guizhou Provincial Engineering Technology Research Center for Chemical Drug R&D, Guizhou Medical University, No. 6 Ankang Road, Guiyang 561113, China

**Keywords:** acute kidney injury, DKS26, ischemia reperfusion, VDR, NF-κB P65

## Abstract

Acute kidney injury (AKI) is a common critical clinical disease with high morbidity and mortality rates. Ischemia-reperfusion (IR) is the main cause of AKI, and there is no effective treatment or prevention. Therefore, it is critical to screen for effective therapeutic agents and to find therapeutic targets. DKS26 is a derivative of oleanolic acid (OA) optimized for bioavailability while retaining the anti-inflammatory, antioxidant, and anti-apoptotic properties of OA. This study aimed to investigate the therapeutic effects of DKS26 on AKI and its underlying molecular mechanisms. We established an AKI model in vivo and in vitro and observed that DKS26 had an ameliorative effect on IR or H/R-induced renal tubular epithelial cell injury and reduced oxidative stress, inflammation, and apoptosis. Meanwhile, Swiss TargetPrediction and AutoDock Vina analysis revealed that DKS26 may interact with vitamin D receptors (VDR) through hydrogen bonding, suggesting that DKS26 may exert effects through VDR. In this study, we found that DKS26 treatment enhanced the stability of the VDR protein, promoted the binding of VDR to *p*-NF-κB P65*^S^^er311^*, reduced the entry of *p*-NF-κB P65*^S^^er311^* into the nucleus, and inhibited the transcription of downstream inflammatory genes as well as their own expression, thus exerting its protective effect. In summary, these findings suggest that DKS26 may be a promising preventive strategy and provide a theoretical and experimental basis for AKI treatment.

## 1. Introduction

Acute kidney injury (AKI) represents a critical global health challenge, with the Global Burden of Disease (GBD) 2021 report estimating 1.3–2.1 million annual deaths worldwide. The prevalence of AKI is reported to be approximately 10–15% in hospitalized patients and up to 50% in ICU patients [1,2]. AKI is characterized as a pathological condition marked by a rapid decline in glomerular filtration rate over a short period, resulting in alterations to kidney physiological function and tissue structure [3]. The causative factors of AKI are recognized to include ischemia-reperfusion (IR), sepsis, and renal transplantation, along with other injuries, with IR being identified as one of the leading causes of AKI. When IR occurs in the kidneys, the oxygen supply is significantly reduced due to a dramatic decrease in blood flow, which subsequently induces oxidative stress, mitochondrial damage, and the production of large quantities of reactive oxygen species as well as pro-inflammatory cytokines, ultimately leading to renal injury [4]. However, no specific or effective treatment has been developed to prevent or treat IR-induced AKI; therefore, the identification of safe and effective therapeutic drugs, in addition to the elucidation of their mechanisms, is considered critical.

The natural product oleanolic acid (OA) is a commonly occurring oleanane-type pentacyclic triterpene with a broad spectrum of anti-inflammatory, antioxidant, and hypoglycemic effects [5,6]. OA, typically found in its free form or as a glycoside, has been extensively studied for its good biological activity [7,8]. Additionally, OA can mitigate acute liver injury induced by chemicals [9], enhance glutathione-mediated antioxidant mechanisms, and alleviate IR-induced myocardial injury [10]. Although OA has achieved certain achievements in alleviating diseases, it is difficult to carry out clinical transformations and applications owing to its low solubility and dose-limiting side effects [11,12]. Therefore, the development of novel OA derivatives with enhanced efficacy and bioavailability is considered critical. This study developed a novel oleanolic acid derivative, DKS26, via C-12,13 hydrogenation and C-28 esterification [13]. Meanwhile, the bioavailability of DKS26 was enhanced via lipid nanoparticle carrier technology, increasing its oral bioavailability from 5.81% to 29.47% [13]. Since the anti-inflammatory and antioxidant effect of it is well-suited to the pathogenesis of IR-mediated AKI, the therapeutic effect was further validated in this model.

Swiss TargetPrediction (http://swisstargetprediction.ch/ (accessed on 18 May 2023)) indicated that DKS26 may act on vitamin D receptors (VDR). VDR is a nuclear receptor belonging to the ligand-activating transcription factor family, and 1,25-(OH)_2_D3 is the main ligand that activates VDR. VDR is expressed in both proximal and distal renal tubular epithelial cells as well as in podocytes. The study found that VDR expression was reduced in both diabetic nephropathy mouse models and renal ischemia-reperfusion rat models, and the administration of VDR activators could alleviate renal ischemia-reperfusion injury in rats [14,15]. Recent studies have shown that VDR can inhibit NF-κB-mediated inflammation and plays a protective role in the progression of kidney disease [16,17]. Mechanistically, IR-induced tissue damage can lead to increased release of inflammatory factors. Inflammatory factors induce Ikk-mediated phosphorylation of nuclear factor kappa B inhibitor alpha (IκBα) and subsequent proteasomal degradation through activation of the TNFR/TLR receptor, which releases Nuclear Factor Kappa-light-chain-enhancer of Activated B Cells (NF-κB) (P65/p50) for nuclear translocation [18,19], and upregulate the expression of pro-inflammatory genes (*TNFα*, *CCL2*), while VDR exerts its protective effect by inhibiting NF-κB P65 nuclear translocation [10]. Therefore, an IR-induced AKI mouse model was used to assess the therapeutic value and possible mechanism of action of DKS26 in AKI. In this study, we found that DKS26 could counteract AKI by inhibiting oxidative stress, improving the inflammatory microenvironment, and attenuating tubular epithelial cell apoptosis. The key effect may be a protective role by stabilizing the VDR and inhibiting the inflammatory pathway of NF-κB P65.

## 2. Results

### 2.1. DKS26 Attenuates IR-Induced Acute Kidney Injury

**Figure 1:**
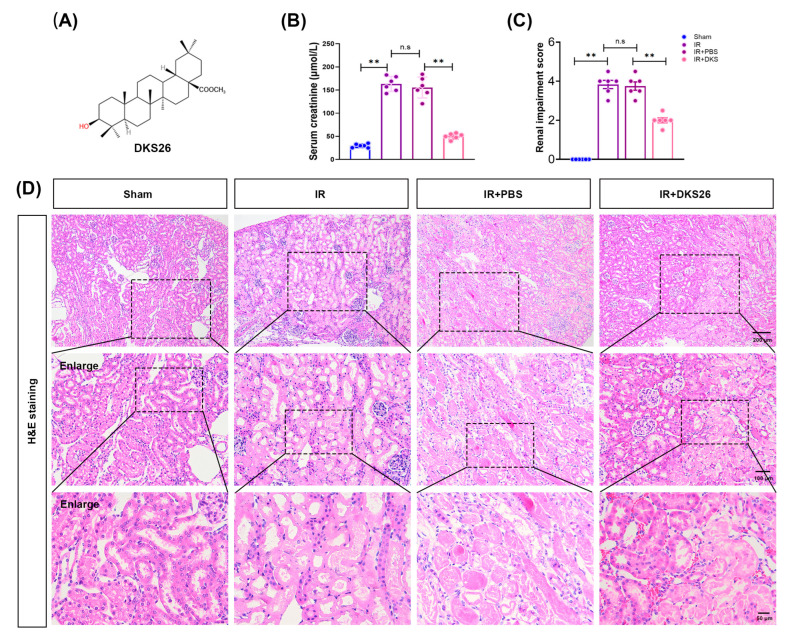
DKS26 attenuated IR-induced tubular infarction and improved renal function. (**A**) Chemical structure of DKS26 small-molecule compound. (**B**) Serum creatinine levels; n = 6. (**C**) Renal injury scores (n = 6). (**D**) Representative H&E staining of kidney tissue. Scale bar: 200 μm, 100 μm, and 50 μm. ** *p* < 0.001 compared to the Sham or IR + PBS groups, with non-significant differences labeled as “n.s”. IR, ischemia-reperfusion; H&E, hematoxylin and eosin.

**Figure 8:**
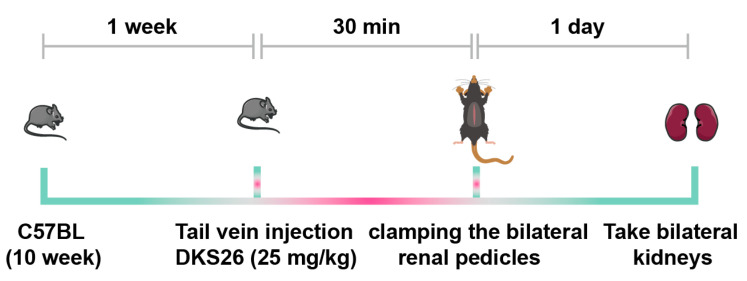
Schematic representation of the timeline for establishing the IR-AKI mouse model and DKS26 administration. DKS26 (25 mg/kg) was administered intravenously via the tail vein, and a 30 min interval was allowed for metabolic distribution in the mice. As previously described, an IR-induced AKI model was established by clamping the renal arteries of C57BL/6 mice for 30 min. IR, ischemia-reperfusion; AKI, acute kidney injury.

DKS26, synthesized from OA via hydrogenation and esterification, retained structural similarity to OA (Figure 1A). Renal functional assessment revealed significantly elevated serum creatinine levels in both the IR and IR + PBS groups compared to the Sham group, which were effectively reversed by DKS26 treatment (Figure 1B; Figure 8). Histopathological analysis revealed extensive tubular infarction at the corticomedullary junction in both the IR and IR + PBS groups compared to the Sham group (Figure 1C,D), characterized by complete necrosis of tubular epithelial cells and loss of cellular architecture. These pathological manifestations were markedly attenuated in DKS26-treated mice, demonstrating their efficacy in improving renal function and alleviating structural injury.

**Figure 2:**
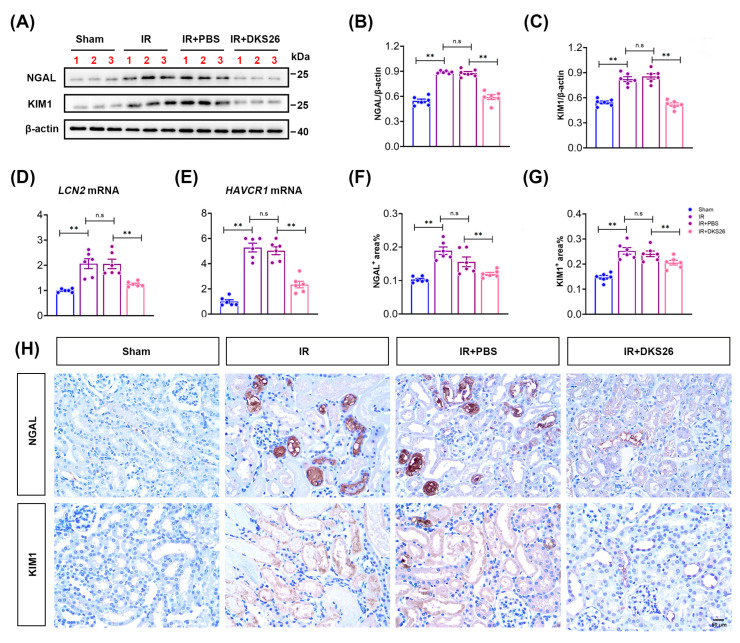
DKS26 treatment reduces the expression of IR-induced kidney injury markers NGAL and KIM1. (**A**) Western blot analysis of NGAL and KIM1 expression in kidney tissues; n = 6. (**B**,**C**) Histograms showing the quantitative analysis of the results from panel A. (**D**,**E**) RT-qPCR analysis of *LCN2* and *HAVCR1* mRNA expression in kidney tissues; n = 6. (**F**,**G**) Analysis of NGAL and KIM1 immunohistochemical staining; n = 6. (**H**) Immunohistochemical staining revealing the localization of NGAL and KIM1 in mouse kidneys; n = 6. Scale bar: 50 μm. ** *p* < 0.001 compared to the Sham or IR + PBS groups, with non-significant differences labeled as “n.s”. IR, ischemia-reperfusion; *LCN2* (encoding NGAL), neutrophil gelatinase-associated lipocalin; *HAVCR1* (encoding KIM1), kidney injury molecule-1; RT-qPCR, real-time quantitative PCR.

The results demonstrated that IR significantly upregulated the protein expression of both neutrophil gelatinase-associated lipocalin (NGAL) and kidney injury molecule-1 (KIM1) in renal tissues. Notably, DKS26 treatment effectively suppressed the IR-induced elevation of these renal injury biomarkers (Figure 2A–C). Consistent with the protein-level findings, RT-qPCR analysis revealed increased mRNA levels of *LCN2* (encoding NGAL) and *HAVCR1* (encoding KIM1) in IR-injured kidneys, which were similarly attenuated by DKS26 administration (Figure 2D,E; Table 1). Immunohistochemical staining showed that NGAL and KIM1 proteins were mainly located in damaged renal tubular epithelial cells. Compared with the Sham group, both the IR and IR + PBS groups exhibited markedly increased positive staining for NGAL and KIM1 (Figure 2F–H). In contrast, the DKS26 intervention substantially reduced the expression of these injury markers (Figure 2F–H). Collectively, DKS26 reduced the expression of renal injury-associated proteins, improved renal function, and attenuated IR-induced renal tubular injury.

### 2.2. DKS26 Inhibits IR-Induced Oxidative Stress, Inflammation, and Apoptosis

**Figure 3:**
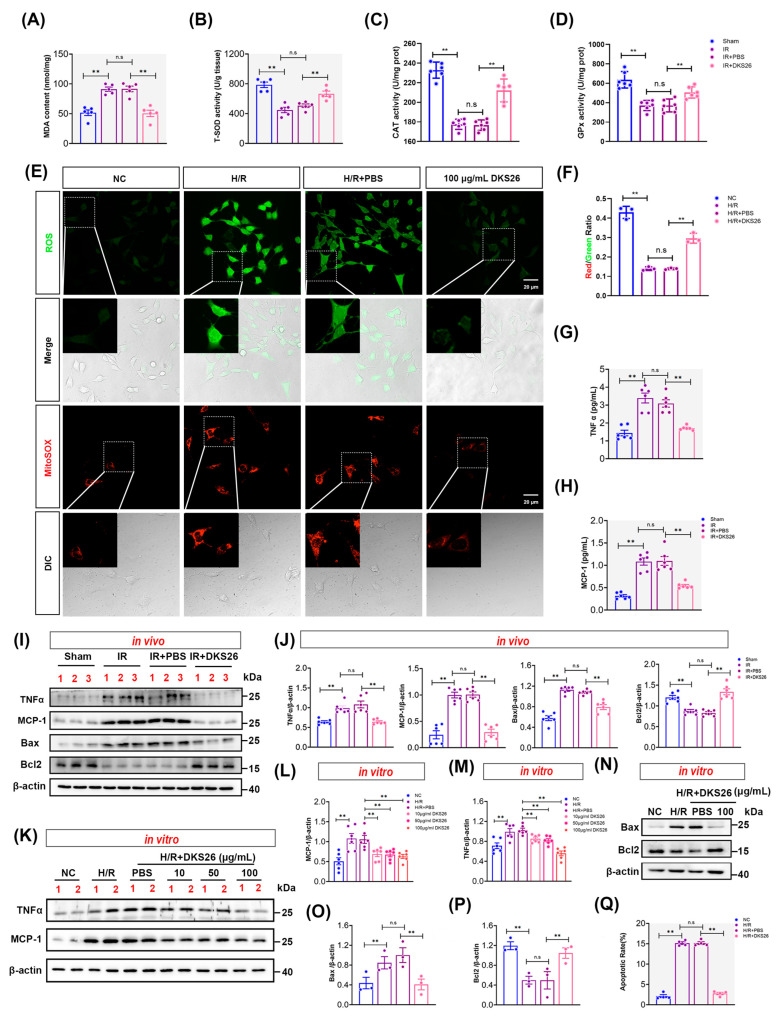
DKS26 improved IR or H/R-induced oxidative and antioxidant imbalance, alleviated inflammation, and inhibited apoptosis. (**A**–**D**) Kit was performed to measure the levels of MDA and the enzyme activities of T-SOD, CAT, and GPx in mouse kidney tissues; n = 6. (**E**) DCFH-DA Fluorescent Probe Staining and Mito SOX Red Staining in mRTECs. Scale bar: 20 μm. (**F**) JC-10 red/green fluorescence staining were quantified using flow cytometry, with a histogram displaying the results; n = 3. (**G**,**H**) ELISA was used to measure the levels of TNFα and MCP-1 in kidney tissue homogenates from each group, n = 6. (**I**) Western blot analysis of TNFα, MCP-1, Bax, and Bcl2 expression in kidney tissues; n = 6. (**J**) Histograms showing the quantitative analysis of the results from panel I. (**K**) Western blot analysis of TNFα and MCP-1 expression in mRTECs; n = 6. (**L**,**M**) Histograms showing the quantitative analysis of the results from panel K. (**N**) Western blot analysis of Bax and Bcl2 expression in mRTECs; n = 3. (**O**,**P**) Histograms showing the quantitative analysis of the results from panel N. (**Q**) Apoptotic cell percentages were quantified using flow cytometry, with a histogram displaying the results; n = 4. ** *p* < 0.001 compared to the Sham or IR + PBS groups in vivo, and ** *p* < 0.001 compared to the NC or H/R + PBS groups in vitro, with non-significant differences labeled as “n.s”. MDA, malondialdehyde; T-SOD, total superoxide dismutase; CAT, catalase; GPx, glutathione peroxidase; TNFα, tumor necrosis factor-alpha; MCP-1, monocyte chemoattractant protein-1; Bax, BCL2-associated X protein; Bcl2, B-cell lymphoma 2; ROS, oxygen species; mRTECs, mouse renal tubular epithelial cells; H/R, hypoxia/reoxygenation; OA, oleanolic acid; IR, ischemia-reperfusion; DIC, differential interference contrast.

In this study, we evaluated cellular antioxidant capacity and oxidative stress status by measuring malondialdehyde (MDA) levels and the activities of total superoxide dismutase (T-SOD), catalase (CAT), and glutathione peroxidase (GPx). Compared to the Sham group, IR induction significantly increased MDA generation in renal tissues and decreased the activities of antioxidant enzymes such as T-SOD, CAT, and GPx, while DKS26 markedly restored the activities of these enzymes (Figure 3A–D), demonstrating that DKS26 enhances cellular antioxidant defense mechanisms. Excessive production of ROS is a primary cause of oxidative stress, and mitochondrial impairment serves as a major intracellular source of ROS. The results demonstrated that hypoxia/reoxygenation (H/R) treatment significantly elevated ROS levels in mouse renal tubular epithelial cells (mRTECs) and reduced mitochondrial membrane potential (Supplementary Figure S1A), whereas DKS26 treatment increased mitochondrial membrane potential, alleviated mitochondrial damage, and thereby effectively suppressed mitochondrial-derived ROS generation (Figure 3E,F). When mitochondrial damage occurs, cells initiate the mitophagy pathway to eliminate impaired mitochondria and maintain cellular homeostasis. Because of that, this study further examined the expression of mitophagy-related proteins. The results demonstrated that H/R stimulation significantly upregulated the expression of PTEN-induced putative kinase 1 (PINK1), Parkin, Microtubule-associated protein 1A/1B-light chain 3-phosphatidylethanolamine conjugate II (LC3 II), and Sequestosome-1 (P62), indicating that mitochondrial injury activates the PINK1/Parkin-dependent mitophagy pathway. After treatment with DKS26, the expression of PINK1 and Parkin was significantly reduced, suggesting that DKS26 may protect mitochondrial structural and functional integrity, thereby decreasing the release of mitochondrial damage signals, while not affecting autophagosome formation or the degradation process of damaged mitochondria (Supplementary Figure S1B). These findings indicate that DKS26 mitigates IR and H/R-induced mitochondrial dysfunction and alleviates oxidative stress.

Tubular infarction and epithelial cell damage are associated with the release of pro-inflammatory factors and macrophage infiltration in IR-AKI models. The expression of the inflammatory cytokines TNFα and MCP-1 was significantly elevated in the IR group. DKS26 treatment attenuated the secretion and release of these inflammatory factors in kidneys (Figure 3G–J). In vitro, H/R promoted the protein expression of TNFα and MCP-1, whereas DKS26 treatment significantly inhibited the expression of these inflammatory factors in a dose-dependent manner, with the 100 μg/mL dose exhibiting the most pronounced effect (Figure 3K–M).

In addition, OA analogues are known to exert antiapoptotic effects. The results showed that both IR and H/R conditions promoted renal tubular cell apoptosis, increased the expression of the pro-apoptotic protein Bax, and decreased the expression of the anti-apoptotic protein Bcl2. DKS26 treatment reversed the imbalance between the pro- and anti-apoptotic factors (Figure 3I,J,N–P). Flow cytometry was further performed to assess cellular apoptosis. Compared with the NC group, the H/R group exhibited an increased apoptotic cell percentage of 15.16%, whereas DKS26 treatment reduced the apoptotic cell percentage to approximately 2.78% (Figure 3Q and Figure S1C). These results collectively demonstrate that DKS26 exerts extensive biological effects, significantly attenuates IR or H/R-induced oxidative stress, inflammation, and apoptosis in mRTECs.

### 2.3. DKS26 Enhances VDR Binding to p-NF-κB P65^Ser311^ to Inhibit NF-κB P65 Nuclear Translocation and Exert Anti-Inflammatory Effects

**Figure 4:**
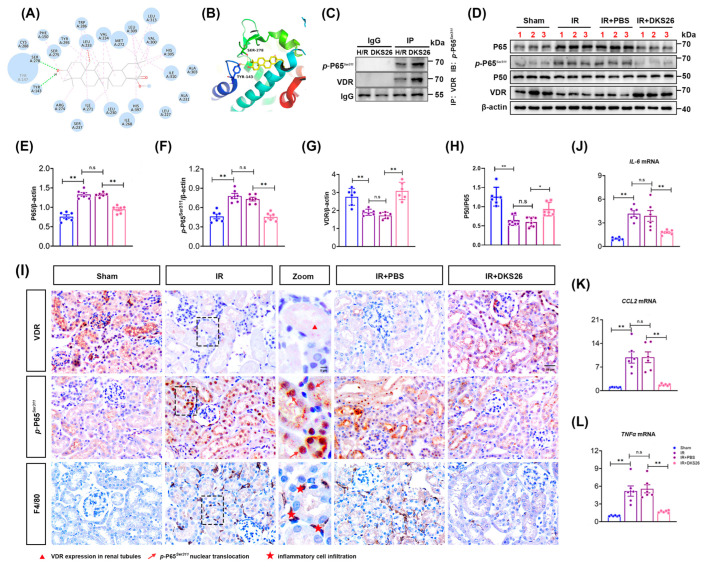
DKS26 inhibited *p*-NF-κB P65*^S^^er^*^311^ nuclear translocation via VDR and reduced the synthesis and secretion of inflammatory factors. (**A**) Molecular docking: two-dimensional binding patterns of DKS26 with VDR. (**B**) Molecular docking: Three-dimensional binding patterns of DKS26 with VDR. (**C**) CO-IP analysis of the interaction between VDR and *p*-P65*^S^^er^*^311^ after DKS26 treatment following H/R. (**D**) Western blot analysis of P65, *p*-P65*^S^^er^*^311^, P50 and VDR expression in kidney tissues; n = 6. (**E**–**G**) Histograms showing the quantitative analysis of the results from panel D. (**H**) Histograms showing the ratio of P50 to P65; n = 6. (**I**) Immunohistochemical staining showing the expression of VDR, *p*-P65*^S^^er^*^311^, and F4/80 in kidney tissues; Zoom represents a locally magnified image of the IR group; n = 6. Scale bar: 50 μm. (**J**–**L**) RT-qPCR analysis of *IL-6*, *CCL2*, and *TNFα* mRNA expression in mRTECs; n = 6. * *p* < 0.05 or ** *p* < 0.001 compared to the Sham, IR, or IR + PBS groups, with non-significant differences labeled as “n.s”. VDR, vitamin D receptor; P65, NF-κB P65 subunit; *p*-P65*^Ser^*^311^, NF-κB P65 phosphorylated at serine 311; P50, NF-κB P50 subunit; IL-6, interleukin-6; *CCL2* (encoding MCP-1), monocyte chemoattractant protein-1; TNFα, tumor necrosis factor-alpha; H/R, hypoxia/reoxygenation; IR, ischemia-reperfusion.

In order to further understand the potential mechanism of DKS26 alleviating IR-AKI, the possible targets of action of DKS26 were predicted on the website (http://swisstargetprediction.ch/ (accessed on 18 May 2023), followed by prioritization of the top five candidate proteins (Supplementary Figure S1D). Among them, VDR is widely expressed in the kidney and is often used as a drug target. It was further confirmed by molecular docking that DKS26 is able to form two important hydrogen bonding interactions with residues SER 278 and TYR 143 on the VDR (Figure 4A,B). Furthermore, several residues, including TYR 295, LEU 233, ARG 274, ILE 271, VAL 234, LEU 309, VAL 330, and HIS 305, contributed to the alkyl and Pi-alkyl interactions (Figure 4A). We finally identified VDR as a possible drug target for further study.

To confirm whether DKS26 affects the NF-κB signaling pathway through the VDR, it was demonstrated by performing the following experiments. In this study, CO-IP experiments confirmed that under H/R conditions, *p*-NF-κB P65*^S^^er311^* could be pulled down by VDR antibodies, indicating an interaction between the two proteins. DKS26 treatment enhanced this interaction, as more *p*-NF-κB P65*^S^^er^*^311^ was pulled down by VDR (Figure 4C). Within the NF-κB signaling pathway, the core subunits NF-κB P50 and P65 exert critical regulatory roles through heterodimer or homodimer formation. In vivo studies showed that IR induced a decrease in VDR expression and increased NF-κB P65 and its phosphorylation in mouse kidneys, without altering NF-κB P50 levels. DKS26 treatment restored VDR expression and suppressed NF-κB P65 and *p*-NF-κB P65*^Ser311^* levels (Figure 4D–H). As the level of NF-κB P50 remains unchanged while NF-κB P65 decreases, this leads to an increased NF-κB P50/P65 ratio (Figure 4H). Immunohistochemical analysis revealed predominant VDR localization in renal tubules, with minimal expression in glomeruli and interstitial regions. IR induced downregulation of renal tubular VDR expression while enhancing nuclear translocation of *p*-NF-κB P65*^Ser^*^311^ and promoting inflammatory cell infiltration. In contrast, DKS26 treatment upregulated VDR expression, suppressed *p*-NF-κB P65*^Ser^*^311^ nuclear translocation, and reversed inflammatory cell infiltration (Figure 4I and Figure S1E–G). RT-qPCR results also showed that IR induced elevated mRNA levels of downstream target genes (*TNFα*, *CCL2*, and *IL-6*) of *p*-NF-κB P65*^Ser^*^311^, whereas DKS26 significantly suppressed the transcriptional levels of these inflammatory mediators (Figure 4J–L; Table 1). These findings suggest that DKS26 ameliorates IR-induced renal injury by modulating the interaction between VDR and *p*-NF-κB P65*^Ser^*^311^, thereby inhibiting the activation of pro-inflammatory signaling cascades.

To confirm whether DKS26 affects the NF-κB signaling pathway through the VDR, it was demonstrated by performing the following experiments. In this study, CO-IP experiments confirmed that under H/R conditions, *p*-NF-κB P65*^S^^er311^* could be pulled down by VDR antibodies, indicating an interaction between the two proteins. DKS26 treatment enhanced this interaction, as more *p*-NF-κB P65*^S^^er^*^311^ was pulled down by VDR (Figure 4C). Within the NF-κB signaling pathway, the core subunits NF-κB P50 and P65 exert critical regulatory roles through heterodimer or homodimer formation. In vivo studies showed that IR induced a decrease in VDR expression and increased NF-κB P65 and its phosphorylation in mouse kidneys, without altering NF-κB P50 levels. DKS26 treatment restored VDR expression and suppressed NF-κB P65 and *p*-NF-κB P65*^Ser311^* levels (Figure 4D–H). As the level of NF-κB P50 remains unchanged while NF-κB P65 decreases, this leads to an increased NF-κB P50/P65 ratio (Figure 4H). Immunohistochemical analysis revealed predominant VDR localization in renal tubules, with minimal expression in glomeruli and interstitial regions. IR induced downregulation of renal tubular VDR expression while enhancing nuclear translocation of *p*-NF-κB P65*^Ser^*^311^ and promoting inflammatory cell infiltration. In contrast, DKS26 treatment upregulated VDR expression, suppressed *p*-NF-κB P65*^Ser^*^311^ nuclear translocation, and reversed inflammatory cell infiltration (Figure 4I and Figure S1E–G). RT-qPCR results also showed that IR induced elevated mRNA levels of downstream target genes (*TNFα*, *CCL2*, and *IL-6*) of *p*-NF-κB P65*^Ser^*^311^, whereas DKS26 significantly suppressed the transcriptional levels of these inflammatory mediators (Figure 4J–L; Table 1). These findings suggest that DKS26 ameliorates IR-induced renal injury by modulating the interaction between VDR and *p*-NF-κB P65*^Ser^*^311^, thereby inhibiting the activation of pro-inflammatory signaling cascades.

### 2.4. DKS26-VDR Complex Formation Enhances VDR Stability, Inhibits p-NF-κB P65^Ser311^ Nuclear Translocation, and Exerts Anti-Inflammatory Effects

**Figure 5:**
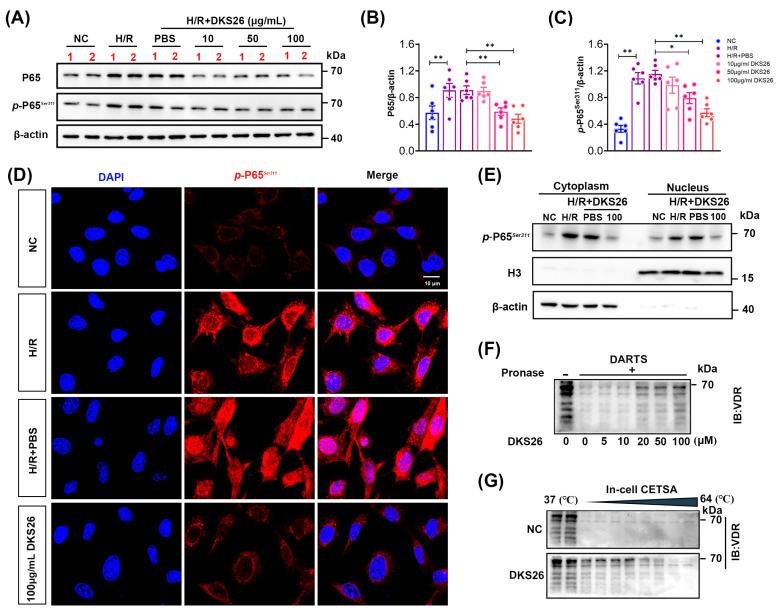
DKS26 forms ligand–protein complexes with VDR to inhibit P65 nuclear translocation and suppress inflammatory responses. (**A**) Western blot analysis of P65 and *p*-P65*^ser^*^311^ expression in mRTECs; n = 6. (**B**,**C**) Histograms showing the quantitative analysis of the results from panel A. (**D**) Confocal laser microscopy showing nuclear translocation of *p*-P65*^ser^*^311^ (red) and nuclear DAPI staining (blue) in mRTECs. Scale bar: 10 μm. (**E**) Cytoplasmic and nuclear fractionation, followed by western blot analysis to detect *p*-P65*^ser^*^311^ in each cell compartment. (**F**) DARTS assay to verify the effect of DKS26 on VDR protein stability. (**G**) Cell thermal shift assay to verify the effect of DKS26 on the thermal stability of VDR. * *p* < 0.05 or ** *p* < 0.001 compared to the NC or H/R + PBS groups. VDR, vitamin D receptor; P65, NF-κB P65 subunit; p-P65*^ser^*^311^, NF-κB P65 phosphorylated at serine 311; H3, histone H3; CETSA, cellular thermal shift assay; DARTS, drug affinity responsive target stability; H/R, hypoxia/reoxygenation.

In vitro experiments demonstrated that DKS26 treatment attenuated the protein expression of NF-κB P65 and *p*-NF-κB P65*^S^^er^*^311^ in a dose-dependent manner, with the most significant therapeutic effect observed at a dose of 100 μg/mL (Figure 5A–C). Fluorescence staining results revealed that H/R induced an increase in nuclear *p*-NF-κB P65*^S^^er^*^311^ expression, while DKS26 intervention significantly inhibited NF-κB P65 phosphorylation and nuclear translocation, suppressing NF-κB pathway activation (Figure 5D). Similarly, cellular fractionation experiments confirmed the protein levels of *p*-NF-κB P65*^S^^er^*^311^ in the cytoplasm and nucleus, yielding results consistent with the fluorescence staining (Figure 5E). Small molecule drugs can enhance protein stability by forming ligand–protein complexes. The DARTS experiment indicated that DKS26 inhibited VDR degradation in a concentration-dependent manner under protease treatment (Figure 5F). Additionally, CETSA demonstrated that DKS26 increased the thermal stability of VDR across a temperature gradient (37–64 °C) (Figure 5G). In summary, DKS26 may bind VDR through hydrogen bonding, enhance VDR protein stability, promote the interaction of the VDR/*p*-NF-κB P65*^S^^er^*^311^ complex in the cytoplasm, reduce the nuclear translocation of free *p*-NF-κB P65*^S^^er^*^311^, and thereby inhibit the NF-κB signaling pathway, exerting anti-inflammatory effects.

### 2.5. DKS26 Exerted VDR Agonist-like Effects and Reversed the Inflammation and Cellular Damage Caused by VDR Inhibitors

**Figure 6:**
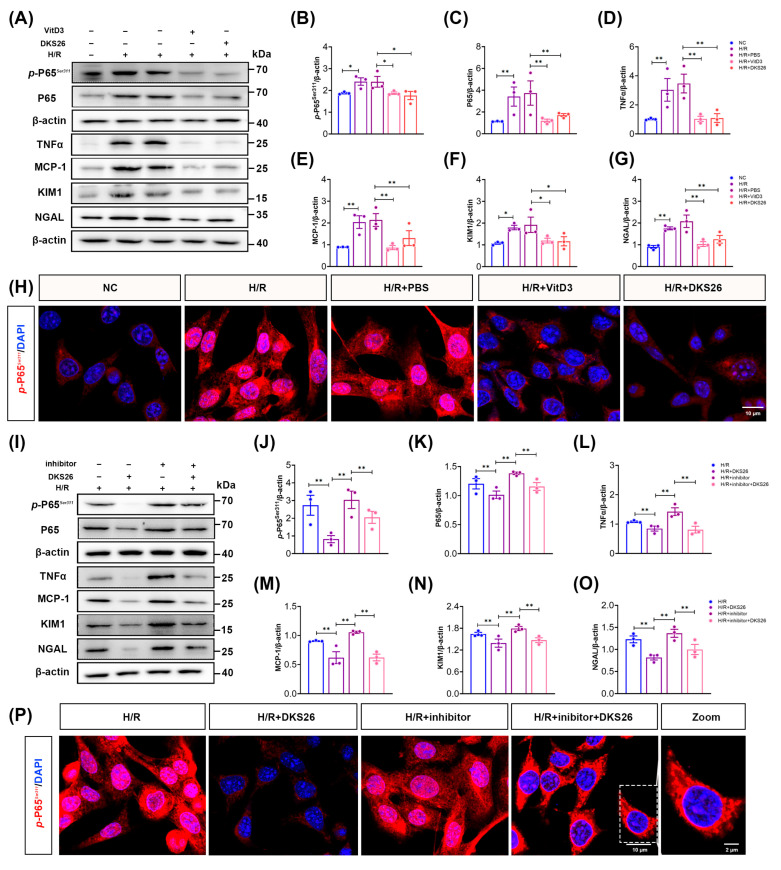
DKS26 partially reversed VDR inhibitor-induced inflammation and cellular damage. (**A**) Western blot detection of *p*-P65*^S^^er^*^311^, P65, TNFα, MCP-1, KIM1, and NGAL expression in cells after administration of the VDR agonist calcifediol and/or DKS26-treated mRETCs; n = 3. Calcifediol: 300 nM, DKS26: 100 μg/mL. (**B**–**G**) Histograms showing the quantitative analysis of the Western blot results from panel A. (**H**) Effects of calcifediol or DKS26 on the nuclear translocation of *p*-P65*^S^^er^*^311^ (red) observed by laser confocal microscopy. Scale bar: 10 μm. * *p* < 0.05 or ** *p* < 0.001 compared to the NC or H/R + PBS. (**I**) Western blot detection of *p*-P65*^S^^er^*^311^, P65, TNFα, MCP-1, KIM1, and NGAL expression in cells after administration of MeTC7 and/or DKS26-treated mRETCs; n = 3. MeTC7: 200 nM, DKS26: 100 μg/mL. (**J**–**O**) Histograms showing the quantitative analysis of the Western blot results from panel I. (**P**) Effects of MeTC7 and DKS26 on the nuclear translocation of *p*-P65*^S^^er^*^311^ (red) observed by laser confocal microscopy; Zoom represents a locally magnified image of the H/R + inhibitor + DKS26 group; Scale bar: 10 μm. * *p* < 0.05 or ** *p* < 0.001 compared to the H/R or H/R + DKS26 groups or H/R + inhibitor. VDR, vitamin D receptor; VitD3, VDR agonist (calcifediol); inhibitor, VDR inhibitor (MeTC7); P65, NF-κB P65 subunit; *p*-P65*^S^^er^*^311^, NF-κB P65 phosphorylated at serine 311; TNFα, tumor necrosis factor-alpha; MCP-1, monocyte chemoattractant protein-1; KIM1, kidney injury molecule 1; NGAL, neutrophil gelatinase-associated lipocalin; H/R, hypoxia/reoxygenation.

To further elucidate the mechanism by which DKS26 exerts its anti-inflammatory effects through VDR, we compared the effects of the VDR agonist (VitD3) and inhibitor (MeTC7) with those of DKS26. The administration of calcifediol reduced cellular *p*-NF-κB P65*^S^^er^*^311^ and NF-κB P65 total protein expression and inhibited downstream inflammatory factor secretion compared with the H/R group, thereby alleviating H/R-induced renal tubular epithelial cell injury; concomitant administration of DKS26 treatment yielded results consistent with calcifediol (Figure 6A–G). And the level of *p*-NF-κB P65*^S^^er^*^311^ nuclear translocation was significantly reduced in cells treated with calcifediol or DKS26 when given at H/R (Figure 6H), suggesting that DKS26 has an effect similar to that of VDR agonists in increasing the activity of VDR. Conversely, administration of the VDR inhibitor MeTC7 promoted phosphorylation of NF-κB P65, facilitated *p*-NF-κB P65*^S^^er^*^311^ nuclear translocation, and increased inflammatory factor synthesis compared with the H/R group, whereas treatment with DKS26 partially reversed the inflammation and cellular damage caused by the VDR inhibitor (Figure 6I–P). In conclusion, DKS26 exerts a VDR agonist-like effect and attenuates H/R-induced renal tubular epithelial cell injury by promoting VDR protein stability and thereby inhibiting the activation of the NF-κB signaling pathway.

### 2.6. DKS26 Also Inhibits NF-κB P65 Activation via the VDR/IKKβ/IKBα Pathway

**Figure 7:**
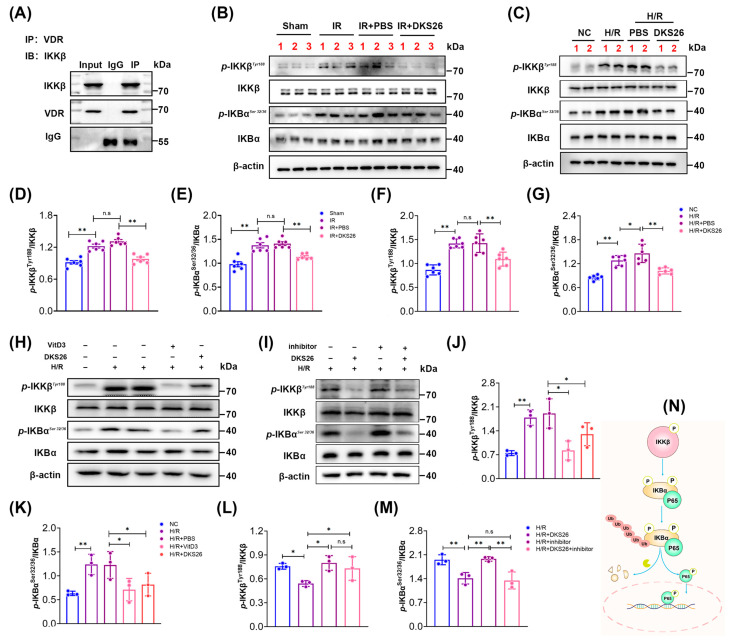
DKS26 inhibited *p*-NF-κB P65*^S^^er^*^311^ activation by modulating IKKβ/IKBα through VDR. (**A**) CO-IP analysis of the interaction between VDR and IKKβ. (**B**) Western blot analysis of *p*-IKKβ, IKKβ, *p*-IKBα, and IKBα expression in kidney tissues; n = 6. (**C**) Western blot analysis of *p*-IKKβ, IKKβ, *p*-IKBα, and IKBα expression in mRTECs; n = 6. (**D**,**E**) Histograms showing the quantitative analysis of the Western blot results from panel B. (**F**,**G**) Histograms showing the quantitative analysis of the Western blot results from panel C. (**H**) Expression of *p*-IKKβ, IKKβ, *p*-IKBα, and IKBα after treatment with the VDR agonist calcifediol or DKS26 in mRTECs; n = 3. Calcifediol: 300 nM, DKS26: 100 μg/mL. (**I**) Western blot analysis of *p*-IKKβ, IKKβ, *p*-IKBα, and IKBα expression in cells treated with DKS26 and/or MeTC7; n = 3. MeTC7: 200 nM, DKS26: 100 μg/mL. (**J**,**K**) Histograms showing the quantitative analysis of the results from panel H. (**L**,**M**) Histograms showing the quantitative analysis of the results from panel I. (**N**) Schematic diagram of IKKβ/IKBα regulation of *p*-NF-κB P65*^S^^er^*^311^ activation. * *p* < 0.05 or ** *p* < 0.001 compared to the Sham/NC or IR/H/R + PBS or H/R + inhibitor groups, with non-significant differences labeled as “n.s”. VDR, vitamin D receptor; IKKβ, inhibitor of nuclear factor kappa-B kinase subunit beta; *p*-IKKβ*^Tyr^*^188^, IKKβ phosphorylated at tyrosine 188; IKBα, nuclear factor kappa B inhibitor alpha; *p*-IKBα, IKBα phosphorylated at serine 32/36; H/R, hypoxia/reoxygenation; IR, ischemia-reperfusion.

It has been found that VDR also interacts with IκB kinase β (IKKβ) to reduce IκBα protein degradation and NF-κB P65/p50 nuclear translocation. IKKβ phosphorylation enhances its activity and promotes IκBα phosphorylation. As an inhibitor of NF-κB P65, phosphorylated IκBα undergoes self-degradation, releasing NF-κB P65, which is subsequently phosphorylated and translocated into the nucleus to mediate its effects (Figure 7N). In this study, CO-IP results confirmed that VDR can interact with IKKβ (Figure 7A). Meanwhile, Western blot results showed that both *p*-IKKβ and *p*-IKBα were increased in the kidney tissues of IR-AKI mice compared with the Sham group; after DKS26 intervention, the phosphorylation of the two proteins was significantly reduced (Figure 7B,D,E). Similar results were observed in vitro (Figure 7C,F,G). In addition, calcifediol reduced the phosphorylation of IKKβ and IKBα under H/R conditions, DKS26 treatment produced similar results as calcifediol (Figure 7H,J,K). In contrast, the VDR inhibitor MeTC7 increased the phosphorylation of IKKβ and IKKα, and the expression of *p*-IKKβ and *p*-IKBα was significantly downregulated by DKS26 intervention on the basis of MeTC7 (Figure 7I,L,M). These results suggest that DKS26 can also indirectly inhibit *p*-NF-κB P65*^S^^er^*^311^-mediated inflammation through the VDR/IKKβ/IKBα signaling axis.

## 3. Discussion

This study investigated the protective effects and underlying mechanisms of DKS26, a novel small-molecule compound, in AKI. Clinical observations indicate that patients with AKI typically exhibit abrupt renal dysfunction marked by elevated serum creatinine levels [20]. In the experimental model, IR-AKI mice developed extensive tubular infarction, increased serum creatinine (Figure 1), and significantly upregulated expression of NGAL and KIM1 at both protein and mRNA levels (Figure 2), confirming renal impairment induced by IR. Treatment with DKS26 markedly reduced tubular injury, and partially recovered renal function (Figure 1 and Figure 2), suggesting its therapeutic potential in alleviating IR-induced AKI.

Previous studies indicate that AKI progression involves tubular epithelial cell damage, accompanied by proinflammatory mediator release [20], macrophage infiltration [4], apoptosis, and oxidative stress [21,22]. While ROS participate in physiological signaling under normal conditions, excessive ROS generation triggers oxidative stress and apoptosis [22]. MDA, combined with antioxidant enzyme activity detection (T-SOD, CAT, GPx), is a standard method for assessing oxidative stress [23]. Thus, this study employed these indicators alongside DCFH-DA and MitoSOX Red staining to quantify intracellular ROS. The results demonstrated that IR and H/R promoted ROS production in both kidney tissue and mRTECs. DKS26 reduced mitochondria-derived ROS, partially restored antioxidant capacity, alleviated oxidative stress, and reversed apoptosis (Figure 3A–F,N–Q). Ischemia/Hypoxia induces mitochondrial damage and activates the PINK1/Parkin-dependent mitochondrial autophagy pathway [24]. In this study, we also found that H/R induced mitochondrial damage and activated the PINK1/Parkin-dependent mitochondrial autophagy pathway; however, the increase in LC3Ⅱ and P62 indicated an increase in autophagosome formation but impaired lysosomal degradation, resulting in ineffective autophagic substrate removal (Supplementary Figure S1B). In addition, Mitochondrial ROS overload promotes Cyto-c release and Caspase-3 activation, upregulating Bax and downregulating Bcl2 [24]. Similarly, this study found that DKS26 may protect the structural and functional integrity of mitochondria, thereby reducing the release of mitochondrial damage signals and preventing cell apoptosis (Figure 3N–Q).

Excessive ROS accumulation in pathological states directly activates the NF-κB pathway, driving inflammatory cytokine synthesis/secretion, renal inflammatory microenvironment formation, inflammatory cell infiltration, and tubular apoptosis [25]. NF-κB activation is pivotal to IR-AKI progression. During IR-AKI, NF-κB-driven inflammatory cytokines (e.g., TNFα) activate Caspase-8, triggering the mitochondrial apoptotic pathway via proapoptotic Bax upregulation [26,27]. Consequently, we further explored DKS26’s anti-inflammatory and antiapoptotic effects. We demonstrated both in vivo and in vitro that both IR and H/R induced the expression of the inflammatory factors TNFα and MCP-1, the pro-apoptotic protein Bax, whereas DKS26 reduced the synthesis and secretion of inflammatory factors, improved the inflammatory microenvironment, and reduced apoptosis (Figure 3). Collectively, DKS26 ameliorated IR and/or H/R-induced oxidative stress, inflammation, and apoptosis.

As a nuclear receptor with renoprotective anti-inflammatory properties, VDR modulates disease progression via direct interaction with NF-κB p65 subunits, as evidenced in disuse muscle atrophy models [14,28]. In the present study, Co-IP assays demonstrated H/R-induced VDR-*p*-NF-κB P65*^S^^er^*^311^ complex formation, which was potentiated by DKS26 treatment (Figure 4C). Functionally, DKS26 restored VDR expression while suppressing *p*-NF-κB P65*^S^^er311^* nuclear translocation and downstream proinflammatory gene transcription and itself (Figure 4 and Figure 5D,E). In vitro H/R models confirmed DKS26’s concentration-dependent inhibition of NF-κB p65 and *p*-NF-κB P65*^S^^er^*^311^ protein expression (Figure 5A–C). Structural stabilization assays revealed that DKS26 enhances VDR stability via ligand–protein complex formation (Figure 5F,G), enabling cytoplasmic sequestration of *p*-NF-κB P65*^S^^er^*^311^ and subsequent blockade of NF-κB transcriptional activity.

By comparing the effects of DKS26 with VDR agonists (VitD3) and inhibitors (MeTC7), we further clarified the mechanism through which DKS26 exerts its renal protective effects by targeting VDR. The results demonstrated that inhibiting VDR activity promotes the synthesis and release of NF-κB P65-mediated inflammatory factors, while both DKS26 and calcifediol reduced the nuclear translocation of *p*-NF-κB P65*^S^^er^*^311^ and suppressed the transcription and synthesis of inflammatory factors. The above suggests that DKS26 exerts VDR agonist-like effects and reverses the inflammation and cellular damage caused by VDR inhibitors (Figure 6). The NF-κB signaling pathway is regulated by the IKKβ/IκBα axis, wherein IKKβ activation phosphorylates IκBα, promoting its ubiquitination and degradation, thereby releasing the NF-κB P65/P50 dimer [29,30]. We demonstrated that VDR directly interacts with IKKβ (Figure 7A) and found that DKS26, along with VDR agonists, reduces IKKβ phosphorylation/activity and decreases IκBα phosphorylation, thereby suppressing NF-κB pathway activation. In contrast, VDR inhibitors exhibited opposing effects (Figure 7B–M). These findings indicate that DKS26 inhibits the activation of the IKKβ/IκBα axis by enhancing the VDR-IKKβ interaction, thereby blocking the transcriptional activity of the NF-κB signaling pathway.

This study systematically elucidates the mechanism by which the novel small-molecule compound DKS26 stabilizes VDR and directly or indirectly inhibits NF-κB nuclear translocation and downstream proinflammatory gene transcription. By orchestrating the inhibition of oxidative stress, inflammation, and apoptosis, DKS26 offers a multi-target therapeutic strategy for IR-AKI. These findings not only highlight the central role of VDR in renal protection but also provide a solid experimental foundation for developing therapies targeting the VDR-NF-κB pathway, demonstrating significant theoretical and translational potential. Although the IR-AKI model is widely used, it may differ from the pathological processes of human AKI, which often involve more comorbidities. DKS26’s efficacy in animal models requires validation in human-relevant systems (e.g., organoids). Future studies should address the lack of pharmacokinetic and safety data by incorporating multiple species, diverse models, and clinical data to fully assess DKS26’s therapeutic potential.

## 4. Materials and Methods

### 4.1. Reagents

VDR (1:500; sc-13133, Santa Cruz, CA, USA), NF-κB P65 (1:500; Sc-8008, Santa Cruz, CA, USA), Phospho-NF-κB P65(*Ser311*) (1:1000; 310299, ZENBIO, Durham, NC, USA), IKKβ (1:1000; 381592, ZEN-BIO, Chengdu, China), Phospho-IKKβ (*Tyr188*) (1:1000; 347345, ZEN-BIO, Chengdu, China), NFKBIA/IkBα (1:500; Sc-373893, Santa Cruz, CA, USA), Phospho-NFKBIA/IkBα (*Ser 32/36*) (1:500; Sc-52943, Santa Cruz, CA, USA), KIM 1 (1:1000; AB233720, Abcam, Cambridge, UK), NGAL (1:1000; #44058, Cell Signaling Technology, Danvers, MA, USA), MCP-1 (1:1000; 220691, ZEN-BIO, Chengdu, China), TNFα (1:1000; 60291-1-AP, Proteintech, Wuhan, China), NF-κB P50 (1:1000; 14220-1-AP, Proteintech, Wuhan, China), PARK2/Parkin (1:1000; 14060-1-AP, Proteintech, Wuhan, China), PINK1 (1:1000; 23274-1-AP, Proteintech, Wuhan, China), LC3A/B (1:1000; #12741, Cell Signaling Technology, Danvers, MA, USA), P62,SQSTM1 (1:1000; 18420-1-AP, Proteintech, Wuhan, China), β-actin (1:10000; 66009-1-Ig, Proteintech, Wuhan, China), HRP-IgG (1:5000; K1223, APEXBIO, Houston, TX, USA), YSFluor™ 594 Goat Anti-Mouse lgG(H+L) (1:200; 33212ES60, YESEN, Shanghai, China).

### 4.2. Experimental Animals

Twenty-four 8-week-old male C57 mice housed in an SPF environment were randomly assigned to four groups: Sham, IR, IR + PBS, and IR + DKS26 (n = 6 per group). The IR-AKI model was induced by clamping the bilateral renal pedicles for 30 min and maintaining ambient temperature at 37 °C. The DKS26 treatment group received DKS26 (25 mg/kg), whereas the PBS control group received an equal volume of PBS, both administered 30 min prior to the clamping procedure. Mice in the Sham group underwent a similar surgical procedure, with kidney exposure but without renal pedicle clamping. The mice were sacrificed 24 h after surgical suture recovery and reperfusion (Figure 8). All animals were treated humanely, and all animal procedures met the relevant legal and ethical requirements according to the protocols (NO:2200404) approved by the Institutional Animal Care and Use Committee of Guizhou Medical University.

### 4.3. Blood Collection, Tissue Preparation, and Biochemical Assays

Following euthanasia, whole blood samples were collected from each experimental group (n = 6 per group) via cardiac puncture. The blood samples were immediately processed by centrifugation at 4 °C and 4000 rpm for 10 min to obtain serum fractions. Scr concentrations were quantitatively determined using a commercially available assay kit (C011-2-1, Nanjing Institute of Biological Engineering, Nanjing, China) in strict accordance with the manufacturer’s protocol. Subsequently, bilateral nephrectomy was performed, and the kidneys were immediately harvested for downstream processing. Renal tissues were precisely sectioned into 1 mm-thick coronal slices using a sterile surgical blade. These tissue sections were allocated for multiple analytical procedures: (1) RNA isolation for gene expression analysis (n = 6 per group), (2) protein extraction for Western blot analysis (n = 6 per group), and (3) paraffin embedding for histological examination (n = 6 per group). The remaining renal tissues were cryopreserved at −80 °C for potential subsequent analyses.

### 4.4. Cell Culture and Drug Intervention

Mouse renal tubular epithelial cells (mRTECs) were purchased from UACC (Tucson, Arizona, USA) and cultured with DMEM (Thermo Fisher Scientific, Waltham, Massachusetts, USA) containing 10% FBS (Thermo Fisher Scientific, Waltham, Massachusetts, USA) in a common incubator with 5% CO_2_ and 95% air with a fusion degree of 70–80% for cell passage or planting into 6-well plates (NEST Laboratories, Pennsylvania, USA) for treatment. (1) The concentration gradient of DKS26 was divided as follows: DKS26 solution was added to 6-well plates at 10, 50, and 100 μg/mL, transferred to an anoxic incubator (94% N_2_, 5% CO_2_, and 1% O_2_) for anoxic culture for 6 h, and then placed under normal culture conditions for reoxygenation for 24 h, and samples were collected for experiments. (2) VDR agonist (calcifediol) /inhibitor (MeTC7) group: Calcifediol 300 nM (MCE, Shanghai, China) or MeTC7 200 nM (MCE, Shanghai, China) were added to the cells in a 6-well plate for 30 min to undergo H/R, and samples were collected for analysis.

### 4.5. Western Blotting

High-efficiency RIPA cracking buffer (PBeyotime, Shanghai, China) was added to the kidney tissues or cells, and a high-throughput tissue grinder was used to grind at a vibration frequency of 50 Hz, and the supernatant was retained by centrifugation. Protein concentration was determined using the BCA method (Elabscience, Houston, TX, USA), and the proteins were separated by SDS-PAGE. After the transfer, the membrane was blocked with 5% skim milk (Yili, Shanghai, China) at room temperature for 1 h. The membrane was washed three times with TBST for 5 min each. The primary antibody was diluted according to the manufacturer’s instructions and incubated with the membranes overnight at 4 °C with gentle shaking. The next day, excess unbound primary antibodies were washed off, and the membranes were incubated with HRP-conjugated secondary antibodies at room temperature. Finally, the images were collected using a chemiluminescence instrument for analysis.

### 4.6. Histopathology and Quantification Analyses

Kidney tissues of 3–4 mm thickness were preserved in 4% paraformaldehyde (Leagene, Beijing, China), dehydrated, embedded, and paraffin sectioned according to staining requirements. Six renal cortical fields were randomly selected for the scoring. The criteria for assessing tubule damage included brush border loss, intraepithelial vacuolar degeneration, cell membrane rupture, nuclear exposure, and an altered tubular structure. Tubular injury was scored as follows: 0, <10% of the tubules were damaged; 1, 10–25% of the tubules were damaged; 2, 25–50% were damaged; 3, 50–75% were damaged; and 4, >75% were damaged.

### 4.7. Immunofluorescence Staining

Cell crawling tablets were fixed with 4% paraformaldehyde for 15 min, washed three times with PBS (Solarbio, Beijing, China), and permeabilized with 0.1% Triton X-100 (Solarbio, Beijing, China) for 10 min. Sections were blocked with 10% donkey serum (Solarbio, Beijing, China)at room temperature for 1 h and incubated with the corresponding primary antibody overnight. The next day, after three washes with PBS, the secondary antibody was added and incubated at room temperature for 1 h, using a film sealing agent containing DAPI (Solarbio, Beijing, China), and observed under a laser confocal microscope.

### 4.8. Immunohistochemistry Staining

Kidney paraffin sections were dewaxed and hydrated with xylene, incubated with 3% hydrogen peroxide at room temperature for 10 min, repaired with 0.1 M sodium citrate microwave oven antigen, sealed with 10% donkey serum, incubated with corresponding antibodies, and refrigerated at 4 °C overnight. The following day, the corresponding secondary antibodies were added, incubated at room temperature for 1 h, and then the ABC enhancer was added and incubated for 30 min. The slides were stained with 3,3′-diaminobenzidine (DAB, zsbio, Beijing, China), counterstained with hematoxylin (Solarbio, Beijing, China), dehydrated, and cover-slipped. Assessment of IHC was scored by applying a semi-quantitative immunoreactivity score.

### 4.9. RT-qPCR

RNA was isolated and extracted from tissues or cells using the TRIzol (Thermo Fisher Scientific, Waltham, Massachusetts, USA) method, and the RNA concentration was determined using a microvolume spectrophotometre. The RNA was reverse transcribed into cDNA using the Fast King gDNA Dispelling RT SuperMix kit (Tiangen, Beijing, China, KR118). cDNA was used as a template for real-time quantitative PCR (RT-qPCR) to detect mRNA expression using the TB Green Premix Ex Taq kit (TaKaRa, Kusatsu, Japan, RR420A). The RT-qPCR reaction protocol was as follows: denaturation at 95 °C for 30 s, annealing at 95 °C for 5 s, and extension at 60 °C for 30 s, repeated for 39 cycles. β-actin was used as the reference gene, and the 2^−ΔΔCt^ method was applied to calculate the relative mRNA expression levels of the target gene.

**Table 1:** Target gene primers.

Gene	Forward (5′-3′)	Reverse (3′-5′)
***HAVCR1 (KIM 1)***	CTGGAATGGCACTGTGACATCC	GCAGATGCCAACATAGAAGCCC
***LCN2 (NGAL)***	ATGTCACCTCCATCCTGGTCAG	GCCACTTGCACATTGTAGCTCTG
***TNFα***	GGTGCCTATGTCTCAGCCTCTT	GCCATAGAACTGATGAGAGGGAG
***IL-6***	TACCACTTCACAAGTCGGAGGC	CTGCAAGTGCATCATCGTTGTTC
***CCL2 (MCP1)***	GCTACAAGAGGATCACCAGCAG	GTCTGGACCCATTCCTTCTTGG

### 4.10. Determination of MDA, T-SOD, CAT and GPx Levels

Mouse kidneys were collected and homogenized in PBS (or normal saline), followed by processing according to the manufacturer’s protocol. Absorbance at 450 nm/405 nm/412 nm was measured using a microplate reader, and MDA levels (Elabscience, Wuhan, China) as well as T-SOD, CAT, and GPx (Nanjing Institute of Biological Engineering, Nanjing, China) activities in tissue homogenates were calculated.

### 4.11. Flow Cytometry

A single-cell suspension was prepared by trypsin digestion of the cells in 6-well plates, and the cell suspension was diluted with 1 × annexin-V binding buffer to a concentration of 1 × 10^6^ cells/mL. Fluorescein isothiocyanate (FITC)-conjugated membrane-associated protein-V and phycoerythrin (PE)-conjugated propidium iodide (PI) (Becton, Dickinson and Company, Franklin Lakes, New Jersey, USA) were added and incubated with the cells at room temperature for 15 min, and dead cells were excluded based on positive PI staining, and live single cells were screened out based on positive FITC staining. The proportion of apoptotic cells relative to the total live cells was calculated, where apoptotic cells included both early and late apoptotic populations.

### 4.12. JC-10 Staining

mRTECs were incubated with 5 μM JC-10 (Solarbio, Beijing, China) at 37 °C for 60 min. A single-cell suspension was generated through trypsinization of cells cultured in 6-well plates. Following two washes with 1× phosphate-buffered saline (PBS), the cells were resuspended in 500 μL of 1 × PBS for subsequent flow cytometric analysis. The fluorescence intensities of red and green channels were quantified, and the corresponding ratios were computed to assess the experimental outcomes.

### 4.13. DCFH-DA Staining

The mRTECs were incubated with 10 μM DCFH-DA (Beyotime, Shanghai, China) at 37 °C for 30 min. The stained cells were washed with PBS. Subsequently, the cells were stained with DAPI for 10 min. Images were acquired using confocal microscopy with excitation light at 488 nm/450 nm.

### 4.14. MitoSOX Staining

The mRTECs were incubated with 5 μM MitoSOX (Thermo Fisher, Shanghai, China) at 37 °C for 45 min. The stained cells were washed three times with PBS. Images were acquired using confocal microscopy with excitation light at 561nm.

### 4.15. Nucleoplasm Separation

The collected cell precipitate was resuspended in buffer A (BestBio, Shanghai, China) pre-supplemented with protease and phosphatase inhibitors and placed on ice for 30 min with shaking once every 10 min. The supernatant (cytoplasmic part) was collected by centrifugation, and extracts B and C, containing protease inhibitors and phosphatase inhibitors, were added to the precipitate. The extracts were then placed on ice for 30 min and shaken every 10 min. After centrifugation, the supernatant (nuclear portion) was collected and stored at −20 °C.

### 4.16. Co-Immunoprecipitation (Co-IP) Assay

The interaction between VDR and NF-κB was detected using a protein A/G magnetic bead immunoprecipitation assay (Elabscience, Wuhan, China). The 5 ng VDR antibody was added to a 40 μg protein sample and incubated overnight in a shaker at 4 °C. The following day, A/G magnetic beads were added to the protein–antibody complex and incubated on a shaker at 37 °C for 2 h. The unbound protein–antibody complex was removed by washing; the formed magnetic bead–protein–antibody complex microbeads were re-suspended in PBS, and SDS sample buffer was added to eluate the bound proteins from the magnetic beads. Western blot analysis was performed using anti-*p*-NF-κB P65*^S^^er311^* antibodies.

### 4.17. Swiss TargetPrediction

The Swiss TargetPrediction website (http://swisstargetprediction.ch/ (accessed on 18 May 2023) was used after inputting the DKS26 chemical structure, forecasting, screening, and its interaction with proteins.

### 4.18. Cellular Thermal Shift Assay (CETSA)

The cells were treated with or without DKS26 for 2 h, lysate was added to crack the cells, and the cell lysates were placed at different temperature gradients (37–64 °C) for 3 min, frozen in liquid nitrogen, and heated to 25 °C after removal. The freeze–thaw cycle was repeated three times, the lysates were centrifuged, and the supernatants were separated and analyzed by Western blotting.

### 4.19. Darts Experiment

The cells were treated for 2 h with or without DKS26, and the cell lysate was diluted with TNC buffer (50 mM Tris-HCl, 50 mM NaCl, 1 mM CaCl2) (1:10). After incubating at room temperature for 30 min, we added 5 μg/mL Pronase and incubated for another 30 min. The reaction was stopped by mixing with loading buffer and analyzed by Western blotting.

### 4.20. Molecular Docking

Molecular docking simulations were performed using the structural template VDR/calcitriol complex deposited in the Protein Data Bank under the code 1DB1 [17]. Typically, the co-crystal ligand VDR was extracted from the crystal complex, and a docking grid was constructed using Autogrid. The AutoDock Vina program was used to dock the processed pdbqt files of DKS26 and the protein, according to previously set parameters.

### 4.21. Statistical Analysis

Statistical analyses were performed using GraphPad Prism v9.0 and IBM SPSS Statistics v24.0. The normality of the data was evaluated using the Shapiro–Wilk test, and normally distributed data are expressed as mean ± standard deviation (SD). Comparisons between two groups were analyzed by an independent samples *t*-test, while multiple group comparisons were assessed using one-way analysis of variance (ANOVA) followed by Tukey’s multiple comparison test. Non-parametric tests are applied if data are not normally distributed. Statistical significance was defined as *p* < 0.05, with non-significant differences labeled as “n.s.”.

## Data Availability

All data needed to evaluate the conclusions in the paper are present in the paper. Additional data related to this paper may be requested from the authors.

## Supplementary Materials

The following supporting information can be downloaded at: https://www.mdpi.com/article/10.3390/ijms26072985/s1.
