# Supplementary material for: DKS26 Alleviates Ischemia-Reperfusion Injury-Induced Acute Kidney Injury by Stabilizing Vitamin D Receptors to Inhibit the Inflammatory Pathway of NF-κB P65
Source: Int J Mol Sci. 2025 Mar 25;26(7):2985. doi: 10.3390/ijms26072985 (PMC11988948; doi:10.3390/ijms26072985)
Supplement: Supplementary file 1 [file ijms-26-02985-s001.zip › ijms-3482891-supplementary.pdf]

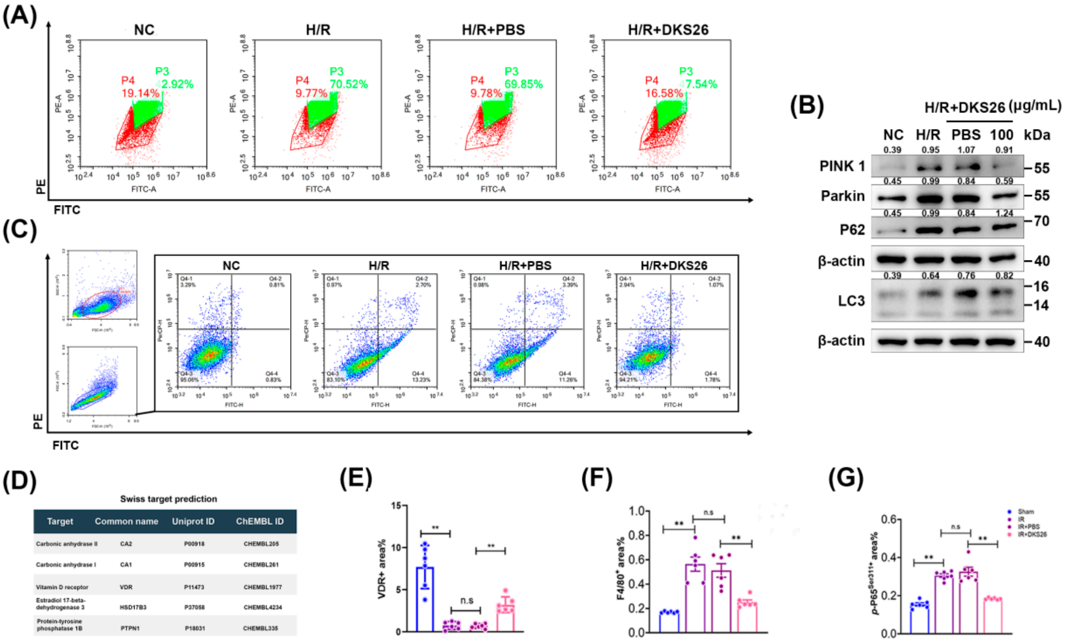

Supplementary Figure S1 (A) Flow cytometry was performed to analyze JC-10 fluorescence staining. **(B)** Western blotting detecting PINK 1, Parkin, LC3 and P62 expression in the mRTECs. **(C)** Apoptotic cell percentages were quantified using flow cytometry. **(D)** Swiss TargetPrediction analysis showing the top five predicted target proteins for DKS26. **(E-G)** Immunohistochemical staining revealing the colocalization of VDR, F4/80, *p*-P65^{Ser311} in mouse kidneys. The results are shown as the mean ± SD; n = 6. **p* < 0.05 compared to H/R group or H/R+PBS group; n.s. indicates no significance. PINK 1, PTEN Induced Putative Kinase 1; Parkin, Parkin RBR E3 Ubiquitin-Protein Ligase; LC3, Microtubule-Associated Protein 1A/1B-Light Chain 3; P62, Ubiquitin-Binding Protein p62.
